# Unleashing the power of colloidal gold immunochromatographic assays for plant virus diagnostics

**DOI:** 10.1016/j.mex.2023.102498

**Published:** 2023-11-29

**Authors:** Abozar Ghorbani, Sajad Astaraki, Mahsa Rostami, Arezoo Pakdel

**Affiliations:** a Nuclear Agriculture Research School, Nuclear Science and Technology Research Institute (NSTRI), Karaj, Iran; b Plant Pathology Department, Faculty of Agriculture, Tarbiat Modares University, Tehran, Iran; c Plant Protection Department, Faculty of Agriculture, Buali Sina University, Hamedan, Iran

**Keywords:** Colloidal gold immunochromatographic assay, ELISA, PCR, Plant virus, Colloidal gold immunochromatographic assays

## Abstract

The colloidal gold immunochromatographic assay (GICA) has become a popular method for the rapid detection of plant viruses. This assay format uses antibodies labeled with colloidal gold to capture and detect specific viral antigens in plant samples. GICA offers several advantages over traditional laboratory-based methods, including speed, ease of use, cost-effectiveness, and accessibility, making it an attractive option for plant virology diagnostics. Because plant viruses can cause significant economic losses in agriculture and horticulture, early detection is essential for effective management and control. Conventional laboratory-based methods such as enzyme-linked immunosorbent assays and polymerase chain reaction are sensitive and specific but require specialized laboratory equipment and training and can be time-consuming and costly. On the other hand, colloidal gold nanoparticles, specific antibodies and carefully designed components are integrated to allow visual detection of target viruses. This makes it an invaluable tool for plant disease management and monitoring that is simple and easy to perform and provides results within minutes. This review article aims to provide a comprehensive overview of the application of colloidal gold immunochromatographic assays for the rapid detection of plant viruses.

## Review Highlights

• GICA is a new novel method to rapidly detect plant viruses by integrating colloidal gold nanoparticles and specific antibodies.
• ELISA and PCR demand expertise and time, GICA provides cost-effective, user-friendly, and quick results.
• The manuscript is a comprehensive guide to GICA's application in plant virus detection, capturing its significance succinctly.

## Graphical abstract

**Figure:**
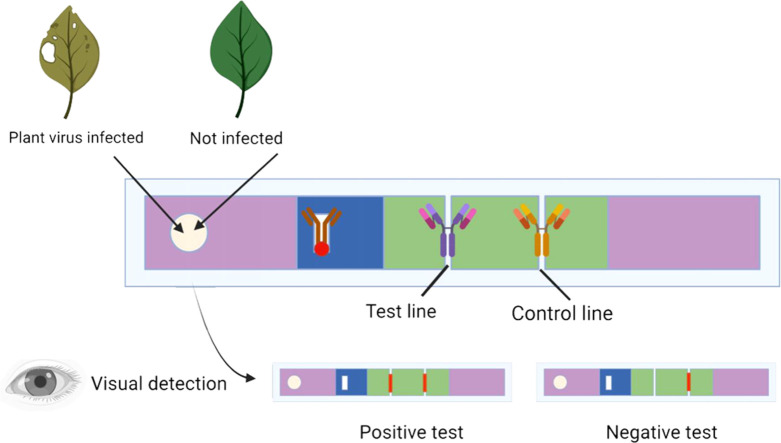

Specifications table

**Table:** 

Subject area:	Agricultural and Biological Sciences
More specific subject area:	Colloidal Gold Immunochromatographic Assays
Name of your method:	Colloidal gold immunochromatographic assays
Name of the reviewed methodology:	Unleashing the Power of Colloidal Gold Immunochromatographic Assays for Plant Virus Diagnostics
Keywords:	Colloidal gold immunochromatographic assay, ELISA, PCR, Plant virus
Review question:	What is the significance of colloidal gold immunochromatographic assays (GICA) in plant virus diagnostics? How does the GICA assay format work in detecting plant viruses? What advantages does GICA offer over conventional laboratory-based methods for plant virus detection? What are the drawbacks of traditional methods like ELISA and PCR for plant virus diagnostics? In what ways does GICA function as an invaluable tool for plant disease management and monitoring?

## Method details

### Introduction

One of the serious cases that highlights the importance of the identification and diagnosis of plant viruses is the globalization of trade and the increase in the human population, which will be accompanied by an increase in the demand for food across several continents of the world. Due to the lack of management and treatment methods for viral diseases, early diagnosis and detection of the pathogens demonstrate the most decisive step to stop the spread of infectious diseases, especially concerning plant viruses [1].

Several methods have been developed for plant viral diagnostics. Initially, identifying disease symptoms by eye was the simplest method to detect viral diseases. In this way, early diagnosis of the disease and the level of damage to the plants could be determined depending on the plant species and its resistance to the virus [2]. During the same period, the inauguration of transmission electron microscopy played a decisive role in the detection and morphological characterization of viral particles. One of the most classic procedures for visualizing viruses in plant tissues was microscopic detection using modern light and high-resolution electron microscopes [3], [4], [5], [6].

Over the last few decades, rapid and specific serological and molecular techniques have been developed for the detection of plant viruses. Serological approaches like enzyme-linked immunosorbent assays (ELISA) for virus detection, which use a specific reaction between an antibody and its corresponding antigen, are among the most important methods for diagnosing and identifying virus diseases. ELISA was first used to detect plant viruses by Clark and Adams in 1977 [7]. Molecular techniques, such as molecular hybridization and DNA amplification, have also been developed for the detection of plant viruses. In the case of hybridization, it is based on binding viral nucleic acids with sequence-specific DNA or RNA probes, due to their sequence complementarity [8]. DNA amplification, which is related to a polymerase chain reaction (PCR), achieves the amplification of many DNA copies of a specific region of the viral genome within a few hours in different cycles and is usually visualized by electrophoresis. The first report on the usage of PCR for the detection of plant viruses can be found in the year 1990 [9].

Researchers were encouraged by the progress of science in the field of nanotechnology to utilize antibody-antigen binding activity in order to develop highly efficient techniques based on immunosensors (The biosensors based on interactions of antigens and antibodies are known as immunosensors) [10]. Gold nanoparticles (GNPs), due to their easy synthesis ability and efficient binding to biomolecules as probes, are a good choice for the development of biosensors in various fields, including the detection of plant viruses. GNPs-based probes have shown more stability, faster response times, and easier results compared to fluorescence-based and enzymatic-based detection systems [11]. The use of gold-labeled IgG complexes for the rapid and specific detection of different viruses in different hosts is described [[12], [13]]. It is noteworthy that the Colloidal gold Immunochromatographic assay (GICA) is based on the specificity of antigen-antibody interactions, which combines colloidal gold labeling and immunochromatography and is currently used as a rapid detection technique [14]. Until now, many studies have shown that this method can be a suitable technique for the detection of viruses that require no laboratory and minimal technical expertise. Additionally, this test can confirm whether a plant is healthy or infected with a particular virus from suspected plant tissues in only 15–20 min [15], [16], [17], [18], [19]. Fast, credible, and precise detection and diagnosis of viruses are necessary to effectively manage and impede the further spread of viral diseases. Most detection methods are time-consuming, difficult to perform, expensive, and require equipped laboratories, making them unsuitable for broad-spectrum field surveys [20]. This study explores the principles, applications, advantages, and limitations of colloidal gold immunochromatographic assays, highlighting their contributions to plant virus detection.

### Colloidal gold immunochromatographic assay: advancing rapid and reliable diagnostics

According to the definition of the International Union of Pure and Applied Chemistry (IUPAC), a biosensor is a device that can utilize a specific biochemical interaction, created by a detection component, to detect and measure a biological molecule [21]. This identification is accomplished by employing an electrical, thermal, or optical signal generated by the transducer. Based on this definition, it can be asserted that every biosensor comprises at least two main components: a biological identifier to interact with the target species, and a transducer to convert the physical or chemical parameters of this biological interaction into a measurable signal. The biological detector layer used in the design of biosensors can be an enzyme, antibody, protein, or DNA. Moreover, the transducers employed in biosensor design can be optical, electrochemical, or based on mass measurement [22].

Immunosensors are a subtype of biosensors in which the biological identifier is an antibody or antigen, and the detection process is the outcome of a specific interaction between this antibody and antigen. Typically, the antibody serves as a component of the identifier and is utilized to identify the antigen as the target species. In the design of immunosensors, like other types of biosensors, the transducers used can be optical, electrochemical, piezoelectric, or calorimetric [23].

**Fig. 1:**
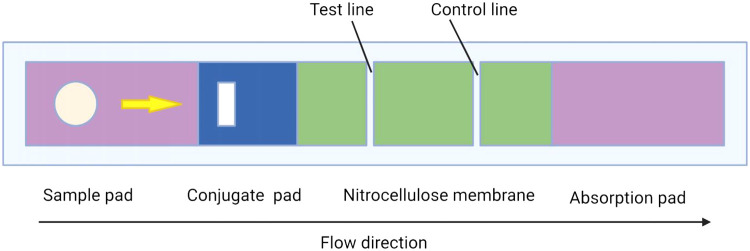
Schematic diagrams of the immunochromatographic test strip.

Among the metal nanoparticles used in protein stabilization, GNPs are the most widely used. In addition to common electrostatic interactions, GNPs can stabilize proteins through covalent bonds between gold atoms and amine and cysteine groups of proteins [[24], [25]]. Methods of Immunochromatographic analysis (IChA) using nanoparticles of colloidal gold as a label have become widespread for analytical purposes, making it feasible to visually detect the compound underdetermination [26]. There are different methods for synthesizing GNPs. Among the chemical methods of gold synthesis, reduction with citrate, the Brust–Schiffrin method and the Micro-Emulsion Method are popular [[27], [28]]. Generally, for the preparation of Colloidal gold particles, the reduction of tetra chloroauric acid with trisodium citrate methods is used [[18], [29], [30]]. GNPs with a size in the range of 3–30 nm exhibit a plasmon strip at ∼520 nm, which red-shifts upon compression or change in mass but is more sensitive to density. Hence, this technique is based on the change in the absorption spectra position of the surface plasmon resonance peak in GNPs, followed by color change [31]. In the case of Colloidal gold-antibody conjugates, usually after purifying the specific monoclonal or polyclonal antibodies (MAb/PAb), the antibodies are added to Colloidal gold solution using different approaches. Consequently, the antigen-antibody reaction can be visualized using Colloidal gold-labeled antibodies [32]. Antibodies labeled with gold particles should be immobilized on an appropriate pad as the reaction substrate or immunochromatographic test strip (ICA). The different parts of ICA strip contain: polyester membrane as a sample pad, a glass fiber membrane as a conjugate release pad, a nitrocellulose membrane, an absorbent paper, and a sticky base [33] (Fig. 1).

**Fig 2:**
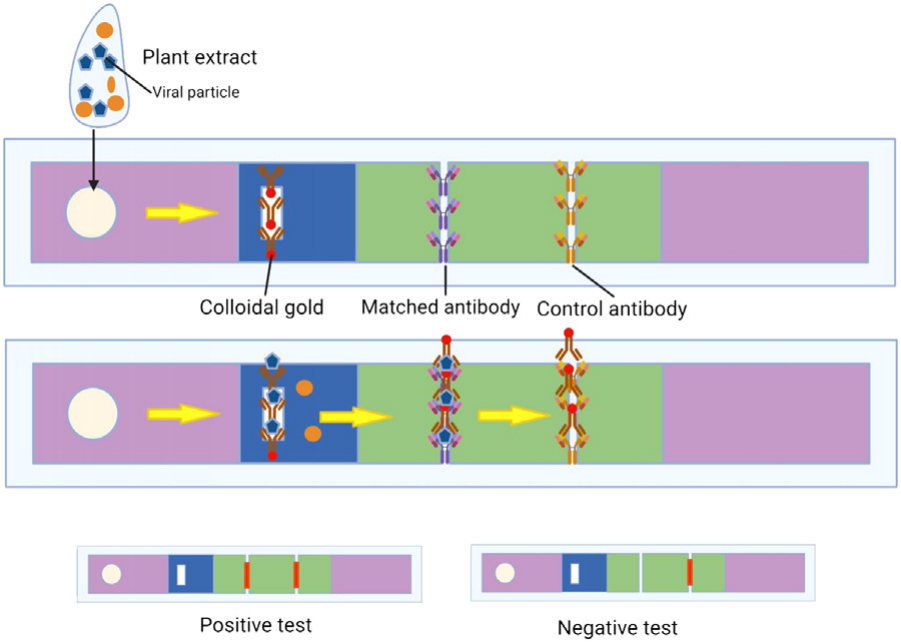
A diagram illustrates the operational principle of the strip. Individual plant extracts are applied to the sample pad. In the event of viral infection within a sample, the antibody + colloidal gold combine with viral particles (depicted as blue dots) at the conjugate pad. This complex migrates towards the nitrocellulose membrane and is captured by a matched antibody at the first band. Simultaneously, unbound antibody + colloidal gold continues through the first line and are captured by a control antibody at the second band. The remaining sample moves onward, accumulating in the absorption pad. The orange dots within the diagram represent distinct plant proteins found in the samples.

The mode of action of this tool is as follows: first, the extract is prepared from the suspected sample, such as infected tissues. Approximately 100 microliters of the extract are then added to the first part. Subsequently, the extract is transferred to the second part, which contains antibodies labeled with gold particles. It is important to note that the direction of the extract flow from the first part is toward the fourth section. In the second part, if the extract contains antigens (viral particles), it reacts with antibodies and gets transferred to the third part. The third part consists of two sections, namely the positive and negative control lines. In this stage, the antibodies that match the specific antigen remain in the control line, while the antibodies that do not have the corresponding antigen function as antigens for the antibodies in the control line (Fig. 2).

Several studies have indicated that optimal pH, colloidal gold particle size, and antibody concentration are crucial factors in enhancing the efficiency of the ICA [[18], [34]].

### The selection and preparation of antibodies and antigens for the colloidal gold immunochromatographic strip assay

The vast diversity of plant viruses, spanning various families, genera, and genomic structures, poses significant challenges for designing effective diagnostic assays and causes selecting appropriate antigens (proteins or nucleic acids) for diagnostic assays can be challenging. Antigens must be specific to the target virus to avoid cross-reactivity with closely related viruses. Cross-reactivity can occur when antibodies for one virus recognize similar components in another virus. This can lead to false-positive results. Some plant viruses exhibit high rates of antigenic variation, making it necessary to continuously update diagnostic reagents to keep up with the evolving virus populations. Antigen selection is a pivotal factor in achieving selectivity in GICA for the detection of plant viruses. [35]. Indeed, the sensitivity and specificity of GICA are highly dependent on the choice and design of the antigen, as well as the quality of generated antibodies [36]. Selectivity, in this context, refers to the assay's ability to accurately distinguish between different viral species, strains, or isolates. Achieving high selectivity is crucial for ensuring that the GICA specifically detects the target virus without cross-reactivity with other related viruses, thereby providing reliable and accurate results. Precisely and purposefully immobilizing antibodies onto gold nanoparticles (AuNPs) ensured that the antigen binding sites were optimally exposed, facilitating their interaction with sugarcane mosaic virus (SCMV) antigens. Consequently, this characteristic enhances the detection of the virus antigens [37].

Choosing the appropriate antigen can be done in four ways:

Specific Antigens: Virus-specific antigens are specific molecular markers or structures that are distinct from a particular virus and are not found in other viruses or organisms. When these unique antigens are used in an assay, it ensures highly specific detection of the target virus and minimizes the likelihood of false positives or cross-reactivity with other pathogens [38]. Researchers have utilized monoclonal antibodies to target unique epitopes on the surface proteins of plant viruses. For instance, in the case of the Potato virus Y (PVY), a highly variable plant virus with multiple strains, researchers have developed monoclonal antibodies targeting distinct epitopes on the PVY coat protein [39]. This allows for the selective detection of different PVY strains in GICA. Another research showed by targeting a specific antigen, PVY and Potato virus X (PVX) were completely and separately identified by lateral flow devices [40]. Moreover, selecting conserved regions or epitopes that are shared among multiple viruses can significantly enhance the assay's ability to detect a wide range of viral strains. This approach leverages the commonalities or similarities present in various viral strains. When conserved regions or epitopes are targeted, the assay is more likely to recognize and bind to a broader spectrum of viruses, even those with minor genetic variations. The using of antibodies to Grapevine leafroll-associated virus 3 (GLRaV-3) that mixture of polyclonal rabbit and monoclonal mouse antibodies caused the recognition of a broad spectrum of GLRaV-3 variants [41].

Conserved Epitopes: Identifying conserved epitopes within the target virus that are less likely to be shared with other pathogens. These epitopes can be used as specific antigenic targets. Expanding research in this area may pave the way for rapid identification of viral infections. For example, common epitopes of Watermelon silver mottle virus have been identified [42]. The choice of which viral component to target as the antigen is crucial. Common options include viral coat proteins, non-structural proteins, or specific epitopes [40]. The most commonly used viruses for peptides as antigens are for example the Cowpea mosaic virus (CPMV), the Tobacco mosaic virus (TMV) and the PVX [43]. Proper purification and preparation of the selected antigen are essential to maintain its structural integrity and antigenic properties. Techniques such as recombinant protein expression, viral particle purification, or chemical conjugation should be optimized for maximum antigen stability [[44], [45]]. Furthermore, Careful antigen design and testing with a panel of relevant samples can help address the issue of potential cross-reactivity with closely related viruses or non-target compounds [46].

Recombinant Antigens: Recombinant antigens can be designed to contain only specific epitopes of interest, minimizing the risk of cross-reactivity with non-target antigens. For example, in the case of Tomato zonate spot virus (TZSV), the recombinant nucleocapsid protein of TZSV was expressed and purified in *Escherichia coli* and used as an antigen to immunize mice. It was used in GICA to distinguish between these closely related tospoviruses [47]. Expression of viral genes is a basic strategy for producing recombinant proteins that can be used for antibody production [48]. The recombinant proteins are then purified as specific antigens for immunization of animals to obtain high-quality virus-specific antibodies [49]. Because of the ease, speed, and cost-effectiveness of producing large amounts of protein with minimal post-translational modifications, *E. coli* is commonly used as a substrate for foreign gene expression [50]. Generally, virions purified from infected plant tissue are used for the production of polyclonal antibodies [51]. The protein from multiple plant viruses is produced in *E. coli* and used to produce virus-specific antibodies for immunological recognition [52], [53], [54], [55]. Also, many recombinant antigens and antibodies can produce in *Nicotiana benthamiana*. This system employs “deconstructed” viral vectors, which are delivered into plant cells via Agrobacterium-mediated systemic delivery to produce recombinant proteins. This system is also effective for generating hetero-oligomeric proteins such as immunoglobulins [56].

Epitope Mapping: Epitope mapping is a technique used to identify the specific regions of an antigen that are recognized by antibodies. By mapping the epitopes of target antigens, researchers can design assays that specifically detect antibodies binding to those epitopes [57]. For instance, Epitope mapping identifies interactions between papaya mosaic virus capsid protein-derived nanoparticles and the immune system [58]. There are several methods for epitope mapping:

Peptide Scanning: This involves synthesizing overlapping peptides that cover the entire antigen and testing them for reactivity with antibodies. Epitopes can be identified based on peptide binding [59].

Alanine Scanning: In this approach, specific amino acids within the antigen are mutated to alanine, and the effect on antibody binding is assessed. This helps pinpoint critical epitopes [60].

Bioinformatics Tools: Bioinformatics plays a crucial role in antigen selection and epitope mapping [61]. Specialized bioinformatics tools can predict antigenic epitopes by analyzing sequence properties, secondary structures, and other features associated with antibody binding. Tools like BLAST can be used to compare sequences and assess potential cross-reactivity. If the 3D structure of the antigen is known or can be predicted, molecular modeling tools can help identify surface-exposed regions that are likely to be antigenic [[62], [63]].

The selection of antibodies for the CGIA directly affects the performance of the assay [64]. The production of antibodies typically involves the immunization of animals (e.g., mice or rabbits) with purified viral proteins or antigens. After the immunization process, antibodies are harvested and purified from the serum. Once antibodies are obtained, they are conjugated or immobilized onto colloidal gold nanoparticles. This step is crucial as it transforms the antibodies into the detection reagent. The conjugation process involves carefully binding the antibodies to the surface of colloidal gold particles while maintaining their activity [[65], [66]]. The conjugation process should be carefully optimized to ensure the stability and functionality of both antibodies and colloidal gold particles. Various methods, such as direct adsorption or covalent binding, can be used to conjugate antibodies with gold nanoparticles. Factors such as antibody concentration, pH, salt concentration, and temperature must be considered to achieve optimal conjugation efficiency and minimize nonspecific binding [67]. Ideally, the antibodies used in the assay should have high affinity and specificity for the target. Monoclonal antibodies are often preferred over polyclonal antibodies due to their consistent quality and well-defined specificity. Monoclonal antibodies offer high specificity due to their single epitope recognition, while polyclonal antibodies can recognize multiple epitopes, enhancing sensitivity [68]. In addition, cross-reactivity with closely related analytes should be minimized to avoid false-positive results. For example, a highly specific and rapid immunochromatographic strip test (ICS) using monoclonal antibodies has been developed for the simple detection of Citrus yellow vein clearing virus (CYVCV) infection [69]. Thorough validation of antibodies by techniques such as ELISA or Western blotting is recommended to ensure their suitability [70]. Rigorous quality control measures should be implemented to ensure the consistency and reproducibility of antigen-antibody interactions. This includes testing the performance of the assay with known positive and negative samples [[71], [72]]. The use of different antigen concentrations to bind antibodies and the comparison with positive and negative samples allowed, for example, rapid and simple detection of potato virus S [73].

### Application of colloidal gold immunochromatographic assay in plant virology

Currently, one of the fastest techniques in the field of identifying plant viruses is the use of GICA [74]. Many studies have shown that this technique is a very suitable approach for the detection of plant viruses (Table 1). The selection of the appropriate assay format is crucial to achieve reliable and sensitive virus detection in plants. Here are the different GICA formats and their importance in the context of plant virus detection:

**Table 1:** Plant virus detection studies with colloidal gold immunochromatographic assay.

Virus name	Host	Ref.
Brome mosaic virus	*Hordeum vulgare*	[12]
Wheat streak mosaic virus	*Triticum aestivum*	
Tobacco mosaic virus	*Nicotiana tabacum*	
Cowpea mosaic virus	*Vigna unguiculata*	
Potato leaf roll virus	*Solanum tuberosum*	
Barley yellow dwarf virus	*Avena sativa*	
Carnation mottle virus	*Dianthus caryophyllus*	[[15], [26]]
Bean mild mosaic virus	*Phaseulus vulgaris*	
Potato viruses X	*Solanum tuberosum*	
Potato viruses Y	*Solanum tuberosum*	
Satsuma dwarf virus	Citrus plant	[65]
Tobacco mosaic virus	*Nicotiana tabacum*	[[15], [26]]
Potato virus X	*Solanum tuberosum*	[75]
Plum pox virus	*Prunus domestica*	
Potato virus X	Potato	[76]
Cucumber mosaic virus	Cucumber	[77]
Lily symptomless virus	*Lilium candidum*	[18]
Soybean mosaic virus	*Glycine* max	[19]
Citrus tristeza virus	Citrus plant	[78]
Large cardamom chirke virus	*Elettaria cardamomum*	[16]
Grapevine leafroll-associated virus	*Vitis vinifera*	[41]
Citrus yellow vein clearing virus	Citrus plant	[69]
Tomato zonate spot tospovirus	*Solanum lycopersicum*	[47]
Rice stripe virus	*Oryza sativa*	[79]
Banana bract mosaic virus	*Musa* sp.	[80]
Tomato brown rugose fruit virus	*Solanum lycopersicum*	[81]
Soybean mosaic virus	*Glycine* max	[82]
Sugarcane mosaic virus	*Saccharum officinarum*	[37]
Sugarcane streak mosaic virus		
Lychnis mottle virus	*Angelica sinensis*	[83]
Plum pox virus	Apricot Tree	[84]
Apple latent spherical virus	*Angelica sinensis*	[85]
Cucumber green mottle mosaic virus	cucumber	[86]

Sandwich Assay Format: In a sandwich GICA, the target plant virus is captured between two specific antibodies, a capture antibody immobilized on the test line and a labeled detection antibody conjugated with colloidal gold. When the virus is present, it forms a "sandwich," resulting in a visible color change. This format is highly specific and sensitive, making it suitable for detecting plant viruses with complex structures and multiple epitopes. It minimizes the risk of false positives and can detect viruses at low concentrations. By using this format, a lateral-flow testing system has been created to swiftly identify PVX in plant leaves, offering a visual detection threshold of 3 ng/mL [[87], [88]].

Competitive Assay Format: The competitive format was designed to identify small molecules that are unsuitable for the sandwich format. In a competitive GICA, a labeled antigen competes with the target in the sample for binding to immobilized antibodies on the test strip. The intensity of the signal is inversely proportional to the sample concentration in the sample. Competitive assays are useful for detecting targets when specific antibodies are limited or unavailable [89]. This format has been used for a wide range of virus types [90] and it can be adapted to detect plant viruses.

Direct Assay Format: In a direct GICA, antibodies labeled with colloidal gold bind directly to the viral antigen in the sample. The presence of the virus results in a visible signal on the test line. Direct assays are simple and rapid and are suitable for the detection of viruses with a single dominant antigenic site [91]. They are particularly useful for on-site testing and point-of-care applications. They have been used for viruses in humans, such as coronaviruses [92]. In addition, this is a common format for the diagnosis of plant viruses by ELISA [93].

Multiplex Assay Format: Multiplex GICA allows the simultaneous detection of multiple plant viruses within a single assay. Different test lines or labels are used for each virus, enabling the detection of several viruses in one test. Multiplex assays are valuable for comprehensive plant virus screening, especially when multiple viruses can infect the same crop. They streamline testing and reduce the need for multiple assays. For example, a multiarray incorporated into a test strip has been designed to identify eight potato pathogens. This technology seamlessly blends the swift detection capabilities of immunochromatography with the high-throughput advantages of array techniques. These pathogens include PVX*,* PVY*,* Potato Virus M, Potato leaf roll virus (PLRV), Necrotic-type potato virus Y, Potato virus S, Potato virus A, and *Clavibacter michiganensis* subsp. sepedonicus. Within the immunochromatographic strip's test zone, meticulously arranged rows of spots contain antibodies tailored to target distinct potato pathogens [[94], [95]].

In the context of plant virus detection, selecting the appropriate GICA format depends on factors such as the specific virus being targeted, the availability of antibodies, the desired sensitivity, the complexity of the sample matrix, and the intended application. Understanding these assay formats and their suitability for different scenarios is crucial for effective plant virus diagnostics and management.

One of the primary works in which gold nanoparticles have been used in the development of rapid detection methods for plant viruses is related to studies that report the use of gold-labeled antibody complexes for the detection of Brome mosaic virus (BMV), Wheat streak mosaic virus (WSMV),TMV, CPMV, PLRV, and Barley yellow dwarf virus (BYDV) [12]. Additionally, this approach was developed to detect TMV [[15], [26]] and PVX [75]. In 2007, a simple and rapid immunochromatographic assay (ICA) was developed to detect the Satsuma dwarf virus (SDV). This assay utilized colloidal gold conjugates of anti-SDV monoclonal antibodies. The study reported that the ICA using the anti-SDV monoclonal antibodies exhibited 8 times and 16 times higher sensitivity compared to the double antibody sandwich-ELISA and ICA using the anti-SDV polyclonal antibody, respectively [65]. In 2010, a method for the detection of Plum pox virus (PPV) using gold-labeled monoclonal antibody conjugates with an optimal component ratio was developed, which included an immunochromatographic express assay (ICA) of PPV that had a detection limit of 3 ng/ml and a duration of 10 min [[18], [75]], Developed an ICA for rapid detection of Lily symptomless virus. The test was based on a double-antibody sandwich format in that the first antibody, was used as the detection antibody conjugated to colloidal gold and the second antibody, was used as the capture antibody at the test line, also they report that the specificity and sensitivity of the ICA were 98.6 % and 100 %, respectively for field leaf samples [19]. Used a highly practical and rapid lateral-flow assay (LFA) for the detection of Soybean mosaic virus. The Lateral-flow immunoassay (LFIA) strip was developed for rapid and cost-effective on-site detection of Citrus tristeza virus (CTV) [78]. Also, lateral flow immunoassay was used field-usable for the rapid detection of Large cardamom chirke virus [16].

### Sensitivity of colloidal gold immunochromatographic assay and its functional difference with ELISA

Methods of immunochromatographic analysis using nanoparticles of colloidal gold as markers are widely used for analytical purposes and allow visual detection of the underdetermined compound [26]. The Limit of Detection (LOD) of a diagnostic assay, such as a GICA, for different targets can vary depending on several factors, including the specific assay design, the quality of reagents, and the target being detected. A lower LOD indicates higher sensitivity and is important for accurately detecting low concentrations of analytes in different applications. LOD is typically expressed in terms of the minimum target concentration that can be reliably detected, often measured in units such as copies/mL or ng/ml [[89], [96]]. The time and LOD in detecting plant viruses with GICA determine its efficiency. For instance, a highly sensitive field immunochromatographic assay was developed for the detection of PVX infection, with a complete assay time of no more than 15 min and the ability to detect at least 2 ng/ml of PVX in non-clarified leaf extract [76]. The rapid immunochromatographic strip described for the detection of CYVCV demonstrated efficiency in detecting CYVCV in tissue extracts at a dilution of 1:320 (w/v), comparable to Dot-ELISA [69]. A monoclonal antibody-based colloidal gold immunochromatographic strip was successfully developed for Tomato zonate spot tospovirus (TZSV), with a visual detection limit of the test strip for TZSV at 800-fold dilutions of TZSV-infected leaf samples [47]. The developed immunochromatographic assay (ICA) for GLRaV-3 detection demonstrated a sensitivity of 100 % and a specificity of 92 % when compared to ELISA as a reference method [41]. Moreover, the ICA could detect Rice stripe virus (RSV) in crude extracts of RSV-infected rice plant tissue diluted to 1:20 (w/v) or 480 µg/mL, as well as in virus homogenate small brown planthopper (SBPH) diluted to 1:2560 (individual SBPH/µL) [79]. A rapid and sensitive lateral flow immunoassay test for the detection of Banana bract mosaic virus in banana plants and their results indicated a very high diagnostic sensitivity (99.04%) and specificity (100%) for the test, which exhibits excellent concordance with ELISA [80]. Furthermore, an ultrasensitive nano-gold-labeled duplex flow immunochromatographic assay was developed for the detection of the SCMV and Sugarcane streak mosaic virus. The linear detection range of the nano-LIFA was 10^–6^ to 10^–9^ g/mL, and with the signal enhancement, the limit of detection reached up to 10^–12^ g/mL [37].

LOD can vary from one GICA to another, even for different plant viruses, due to differences in the antibodies or antigens used, assay conditions, and the format of the assay. For example, the increased affinity of the anti-PPV compared with primary antibodies was investigated and studied several times, and finally, this test was appropriate for the virus [75]. Also, the LOD may differ depending on the specific virus targeted, as some viruses have unique properties or characteristics that facilitate or complicate their detection. This may also be influenced by the type of sample matrix (e.g., leaf extracts, sap, or soil), as the matrix can affect the efficiency of virus extraction and assay performance. The colloidal gold immunochromatographic strip was used to detect Tomato brown rugose fruit virus (ToBRFV) and the sensitivity test results showed that according to the sensitivity test outcomes, it was determined that the minimum detection threshold for ToBRFV-infected tomato leaf extracts was 12,800 times, while for ToBRFV particles it was 50 ng [81].

The LOD can be improved by using some techniques. For example, a straightforward method was devised to enhance the sensitivity of the sandwich lateral flow immunoassay by modifying the binding conditions between a polyvalent antigen and gold nanoparticle-antibody conjugates. In this investigation, the polyvalent antigen employed was the PVY, while the antibodies were coupled with gold nanoparticles measuring 17.4 ± 1.0 nm in diameter. The dried GNP conjugates were applied on the test strip both before and after pre-incubation with the sample. Interestingly, a 30-second pre-incubation of the GNP conjugates and the sample led to a remarkable 60-fold improvement in the detection limit, reducing it from 330 ng∙mL−1 to just 5.4 ng∙mL−1 compared to the conventional lateral flow immunoassay. The quantitative outcomes of this enhanced lateral flow immunoassay, involving pre-incubation, were substantiated via ELISA, yielding a robust correlation coefficient of 0.891 [97]. Moreover, a coronavirus disease detection study showed that EDTA-K2 enhanced the positive antibody signal by chelation with colloidal gold and improved the detection sensitivity of SARS-CoV-2 IgM and IgG antibodies when GICA was used [98]. Another way to solve the problem of LOD in lateral flow strips is to use CRISPR technology in strips. The CRISPR diagnostic method can also be performed on lateral flow strips but is based on the extraction of nucleic acids, not antibodies and antigens so this method is completely different from serological tests [99].

### Advantages of the colloidal gold immunochromatographic assay for the detection of plant viruses

Rapid Results: One of the primary advantages of colloidal gold immunochromatographic assays is their speed. They provide quick results within minutes, enabling rapid screening and detection of plant viruses [47]. Compared to traditional methods like ELISA, which can take hours to complete, GICA provides a significant time advantage and in many cases, they have similar accuracy [100]. This is particularly important in agricultural settings where time is of the essence to prevent the spread of diseases. Many studies have demonstrated the feasibility of using GICAs for the detection of various plant viruses (Table 1). For instance, the urgent requirement for detecting the Apple latent spherical virus (ALSV) is crucial for the ongoing advancement of the angelica industry. Consequently, it is imperative to create straightforward, highly sensitive, and dependable methods for detecting ALSV. To address this need, a rapid detection system employing GICA has been developed and fine-tuned for the detection of ALSV. This GICA assay has proven effective in identifying ALSV in infected plants, with no instances of cross-reactivity observed with three other plant viruses. The sensitivity of the GICA method matches that of the GICA-RT-PCR. Significantly, this approach is capable of detecting and distinguishing ALSV under field conditions [85].

User-Friendly: These assays are designed to be user-friendly and do not require specialized laboratory equipment or technical expertise. They are simple to use and can be performed in the field, allowing for on-site testing without the need for complex laboratory infrastructure. Methods like PCR and sequencing require specialized equipment and trained personnel, whereas GICA can be used by non-specialists [[69], [77]]. For example, GICA is a cost-effective approach for use in detecting the Lychnis mottle virus without extracting nucleic acids [83].

Portability: Colloidal gold immunochromatographic assays are portable and can be easily carried to different locations. This mobility is advantageous for surveillance purposes, as it allows for efficient monitoring of plant virus outbreaks across various fields or regions [37]. PCR and sequencing typically require laboratory settings and equipment, limiting their application in remote or field environments. A straightforward and easily transportable system has been outlined for swiftly identifying Grapevine fanleaf virus in infected plants. This method utilizes immunofiltration, followed by magnetic detection and quantification of virus particles labeled with magnets [101].

Visual Interpretation: The results of these assays are typically interpreted visually, often as colored lines or signals [102]. This eliminates the need for sophisticated analytical instruments and enables rapid on-site diagnosis without the requirement of skilled personnel or access to a laboratory. These assays exhibit greater sensitivity and shorter processing times when contrasted with traditional PCR methods, with results that can be readily interpreted through visual means [103].

Sensitivity and Specificity: Colloidal gold immunochromatographic assays have been developed to provide high sensitivity and specificity in the detection of viruses. They utilize antibodies or other specific binding agents that recognize viral antigens, ensuring accurate identification of target viruses while minimizing false positives or negatives [88]. The ICS test, employed for detecting citrus yellow vein clearing virus in field samples, underwent a rigorous assessment of its specificity and sensitivity. Remarkably, it demonstrated no cross-reactivity with other pathogens, including CTV, SDV, Citrus tatter leaf virus, Citrus exocortis viroid, Citrus mosaic virus, Citrus psorosis virus, *Citrus ringspot virus*, or *'Candidatus* Liberibacter asiaticus*'*. Furthermore, the ICS test successfully identified CYVCV in tissue extracts at a 1:320 (w/v) dilution, matching the efficiency of the dot-ELISA assay. In summary, the ICS assay offers a cost-effective, faster, and simpler alternative to conventional CYVCV detection methods, making it a valuable tool for large-scale CYVCV detection or monitoring efforts [69].

Cost-Effectiveness: Compared to more complex laboratory-based techniques, colloidal gold immunochromatographic assays are generally more cost-effective. They offer a relatively inexpensive solution for rapid screening and surveillance, making them accessible to a wider range of users, including farmers, agronomists, and plant health inspectors. PCR and sequencing can be expensive due to the cost of reagents and specialized equipment, making GICA a more budget-friendly option for routine monitoring [104].

Multiplexing Capability: GICA can be adapted for multiplexing, enabling simultaneous detection of multiple plant viruses in a single test, which is essential for comprehensive screening. Multiplexing with PCR can be complex and requires careful primer design and optimization. A Triplex Lateral Flow Immunoassay has been created for the swift diagnosis of three viruses: TMV*, Tobacco Vein Banding Mosaic Virus* (TVBMV), and PVY. This test boasts limits of detection, reaching 200 ppb (ng/ml) for TMV, 1 ppm (µg/ml) for PVY, and 2 ppm for TVBMV particles [105].

It's worth noting that while colloidal gold immunochromatographic assays have numerous advantages, they also have limitations.

### Disadvantages of the colloidal gold immunochromatographic assay for the detection of plant viruses

Sensitivity: Colloidal gold immunochromatographic assays may have lower sensitivity compared to other more sophisticated laboratory techniques like PCR or ELISA. This means that the assay may not be able to detect low levels of the virus, leading to false-negative results [106]. ICA was designed for rapid detection of GLRaV-3 using a sandwich immunoassay that examined three different preparations of AuNPs. The study showed that AuNPs with a maximum average diameter were most effective in detecting GLRaV-3 at the highest dilutions. The assay exhibited excellent sensitivity, achieving a detection rate of 100 % and a remarkable specificity of 92 % compared to ELISA. Furthermore, the ICA showed a sensitivity of 93 % compared to PCR and maintained the same high specificity of 92 %. Although this result shows the high potential of ICA, PCR and ELISA provide the complete test result [107].

Specificity: The assay's specificity, or the ability to accurately detect the target virus without cross-reacting with other substances, can be a concern. Depending on the design and quality of the antibodies used, there is a possibility of false-positive or false-negative results due to non-specific binding or cross-reactivity with similar viruses or substances [35].

Detection Limit: The detection limit of colloidal gold immunochromatographic assays may not be as low as other molecular-based methods. This can limit their usefulness in detecting viruses present at very low concentrations [84]. So, GICA may not be suitable for detecting viruses at extremely low concentrations, limiting its use for early disease detection. PCR and real-time PCR can detect viruses at very low concentrations, making it more suitable for early detection. An ICS test for specific detection of Lily symptomless virus was developed. The result showed the specificity and sensitivity of the ICS compared to the RT-PCR was lower [18].

Cross-Reactivity: Some GICA assays may exhibit cross-reactivity with closely related viruses or substances, potentially leading to misinterpretation of results, but another method such as PCR can be highly specific when properly designed, reducing the risk of cross-reactivity. To evaluate the efficacy of GICAs, one of the tests required is a cross-reactivity test. All tests performed after demonstrating that there is no cross-reactivity with closely related viruses of the target virus will be publicly presented and made available [[94], [108]].

## Conclusion

The colloidal gold immunochromatographic strip assay has emerged as a powerful and efficient tool for the rapid detection of plant viruses. This review has provided a comprehensive overview of the various aspects associated with this assay, including its principles, components, advantages, limitations, and applications. Importantly, the colloidal gold immunochromatographic strip assay has demonstrated significant potential for on-site testing in field conditions, enabling early detection and rapid response to plant virus outbreaks. Its simplicity and cost-effectiveness make it particularly suitable for resource-limited settings, where sophisticated laboratory facilities may be lacking. While the assay has proven successful in detecting numerous plant viruses, further research and development are necessary to address certain challenges, such as enhancing the assay's sensitivity for low titer infections and improving its specificity to minimize cross-reactivity. Additionally, the integration of emerging technologies, such as smartphone-based detection and digital image analysis, holds promise for further enhancing the assay's convenience and accuracy. Overall, the colloidal gold immunochromatographic strip assay is a valuable and versatile tool for rapid plant virus detection. Its potential applications in plant disease management, quarantine measures, and crop protection make it an essential technique for safeguarding agricultural productivity and ensuring food security. Continued research and innovation in this field will undoubtedly contribute to the development of even more advanced and efficient diagnostic tools, ultimately benefiting the agricultural industry and global food production.

## Ethics statements

Not applicable.

## CRediT authorship contribution statement

**Abozar Ghorbani:** Supervision, Conceptualization, Methodology, Data curation, Writing – review & editing, Visualization, Investigation. **Sajad Astaraki:** Data curation, Writing – original draft. **Mahsa Rostami:** Data curation, Writing – review & editing. **Arezoo Pakdel:** Data curation, Writing – review & editing.

## Declaration of Competing Interest

The authors declare that they have no known competing financial interests or personal relationships that could have appeared to influence the work reported in this paper.

## Data Availability

No data was used for the research described in the article.
